# Clinical Manifestations, Antifungal Susceptibilities, and Outcome of Ocular Infections Caused by *Purpureocillium lilacinum*

**DOI:** 10.3390/microorganisms13122858

**Published:** 2025-12-16

**Authors:** Xinlei Zhao, Jinliang Jiang, Huijing Huang, Jiayi Zheng, Liuxueying Zhong, Fang Duan

**Affiliations:** State Key Laboratory of Ophthalmology, Zhongshan Ophthalmic Center, Sun Yat-sen University, Guangzhou 510060, China; xinleizhao2000@163.com (X.Z.);

**Keywords:** *Purpureocillium lilacinum*, antifungal susceptibility, oculomycosis, visual outcomes, voriconazole

## Abstract

*Purpureocillium lilacinum* is an emerging pathogen that can cause severe ocular infections. This study aimed to investigate the risk factors, clinical manifestations, antifungal susceptibilities, and outcomes of ocular infections caused by *P. lilacinum* at a large ophthalmic center in Southern China. This retrospective study reviewed the medical records of 34 patients with culture-proven *P. lilacinum* oculomycosis treated at the Zhongshan Ophthalmic Center from January 2020 to December 2024. The study included 34 patients (17 males, 17 females). The most common risk factor was ocular trauma (38.2%). In vitro susceptibility testing revealed high resistance to fluconazole and caspofungin, but general susceptibility to voriconazole (median MIC 0.25 mg/L). Despite 97.1% of patients receiving voriconazole therapy, outcomes were generally poor, with 54.5% of patients experiencing a poor outcome (vision worse than counting fingers). A significantly shorter time to microbiological diagnosis was associated with a favorable outcome (median 26 days vs. 65 days, *p* = 0.007). In conclusion, the visual outcomes of this infection remain generally poor, with the major clinical challenge being the delay in diagnosis. Therefore, prompt microbiological investigation is recommended for patients with suspected intraocular infection. Voriconazole remains the first-line therapeutic choice, the therapeutic potential of newer triazoles warrants further investigation.

## 1. Introduction

Oculomycosis refers to a group of fungal infections affecting the eye and its associated structures. Corneal infection (Keratitis) is the most frequent presentation, but the orbit, eyelids, lacrimal apparatus, conjunctiva, sclera, and intraocular structures (endophthalmitis) can also be affected [1]. It is estimated that fungal keratitis affects over one million individuals annually, with 8–11% of the patients losing the eye [2]. The spectrum of fungi involved varies based on geographic origin, socioeconomic level, and climatic conditions [3]. In general, filamentous fungi account for the majority of infections in tropical and subtropical regions, whereas yeast infections are more common in temperate zones [2,3].

*Purpureocillium lilacinum* (*P. lilacinum*, formerly *Paecilomyces lilacinus*), is a globally distributed filamentous fungus inhabiting diverse environments such as soil, grasslands, and even within insects [4,5]. It has been widely investigated as a promising biocontrol agent against plant-parasitic nematodes because of its production of Leucinostatins [6,7]. However, the pathogenic mechanisms underlying its infection in humans remain unclear. Previous studies have indicated that Conidia of *P. lilacinum* can infect and destroy macrophages as well as dendritic cells, providing clear evidence that this pathogenic fungus is capable of invading human phagocytic cells [8]. A retrospective study on *P. lilacinum* infection indicated that oculomycosis is the most common manifestation, followed by cutaneous and subcutaneous infections and a smaller proportion of infections at other sites [9]. A literature review revealed 119 documented cases of human infection caused by *P. lilacinum* from 1964 to 2004, with 61 instances involving oculomycosis. The most common predisposing factor was lens implantation, and 20 cases developed into eye enucleation [9]. Yuan et al. reported 17 cases of *Paecilomyces* keratitis from two referral centers in the United States in 2009, and they found that the most common risk factor was a preexisting corneal disease or previous ocular surgery [10]. Ali et al. reported 28 patients with *P. lilacinum* keratitis at the Bascom Palmer Eye Institute from 2007 to 2013, and found that over 70% of the patients had been wearing soft contact lenses [11]. Turner et al. reported 21 cases of *P. lilacinum* oculomycosis from 2000 to 2012 in Australia and found the primary risk factor in their cases was immunosuppression [12]. Only several cases have been reported in mainland China to date [13,14]. Chen et al. reported 12 cases with *P. lilacinum* keratitis between 2003 and 2017 in Taiwan. All of these studies revealed that *P. lilacinum* oculomycosis caused ingressive visual impairment, even eye enucleation.

Previous limited studies on *P. lilacinum* oculomycosis had a relatively small sample size. Furthermore, risk factors and clinical manifestations of infections vary across regions and over time. Therefore, we retrospectively reviewed the clinical and microbiological features of 34 patients with culture-proven *P. lilacinum* oculomycosis at Zhongshan Ophthalmic Center in southern China from 1 January 2020, to 31 December 2024, to investigate the specific risk factors, clinical manifestations, antifungal susceptibility, treatment options, and visual outcomes of this emerging pathogen.

## 2. Materials and Methods

This research involved a retrospective analysis of electronic medical records from patients diagnosed with culture-confirmed *P. lilacinum* oculomycosis at Zhongshan Ophthalmic Center from 1 January 2020 to 31 December 2024. Data collected included demographic details, medical history, slit-lamp examination results, risk factors, initial visual acuity, therapeutic interventions, antifungal susceptibility, and final visual acuity. Endophthalmitis was clinically defined by evidence of inflammation in anterior and posterior ocular segments during ophthalmologic evaluation [15]. All cases of endophthalmitis in this study exhibited vitritis, confirmed either through clinical assessment or detection of vitreous opacities via B-scan ultrasonography. This study was performed in compliance with the principles of the Helsinki agreement and was approved by the Institutional Ethics Committee of Zhongshan Ophthalmic Center, Sun Yat-sen University. The requirement for patient consent was waived given the retrospective nature of the study.

### 2.1. Pathogen Isolation and Identification

Specimens were collected in the hospital’s microbiology laboratory under local anesthesia (0.5% proparacaine hydrochloride) or intraoperatively, using procedures based on previously published protocols [16]. Corneal specimens were collected by scraping the base and periphery of the pathological lesions, while anterior chamber fluid and vitreous bodies were extracted via syringe aspiration or through vitrectomy procedures performed in the operating room. The collected specimens were tested by direct smears and culturing. A portion of each specimen was examined microscopically for the presence of fungi, bacteria, or *Acanthamoeba* by staining with fungal fluorescence stain or Gram stain. Another portion was subjected to fungi, bacteria, or *Acanthamoeba* culture. Fungal culture was conducted on potato dextrose agar at 28 °C for a period of seven days. Identification of the fungal genus was performed by medical technologists based on colony morphology observed on potato dextrose agar, as well as microscopic examination of hyphal and spore structures using lacto-phenol cotton blue staining. Simultaneously, the isolates were subjected to identification using the VITEK MS MALDI-TOF MS system (bioMérieux, Marcy-l’Étoile, France; database version IVD KB3.2).

A mold sample of approximately 1 cm^2^ was treated with 900 μL of 70% ethanol for inactivation, then centrifuged at 14,000× *g* for 2 min. The supernatant was discarded, and the pellet was resuspended in a mixture of 40 μL of 70% formic acid and 40 μL of acetonitrile to facilitate protein extraction. Following a second centrifugation at 14,000× *g* for 2 min, 1 μL of the resulting supernatant was applied onto a target plate, permitted to dry naturally, and subsequently covered with 1 μL of a saturated α-cyano−4-hydroxycinnamic acid (CHCA) matrix solution. After air-drying, the samples were subjected to analysis. Mass spectra within a range of 2000 to 20,000 Da were recorded in linear positive mode at a frequency of 50 Hz by a 337 nm nitrogen laser with a fixed focus. Testing was conducted in duplicate.

### 2.2. Antifungal Susceptibility Testing

Following the identification of fungal isolates, susceptibility testing for voriconazole, caspofungin, and fluconazole was conducted using the E-test method in accordance with CLSI guidelines. Commercially available test kits (Autobio Diagnostics Co., Ltd., Zhengzhou, China), approved by the China Food and Drug Administration, were utilized. Susceptibility assays were carried out using pure fungal colonies. Plates were inoculated in three directions with a nontoxic cotton swab dipped in the diluted stock inoculum suspensions. Then, appropriate E-test strips were placed onto the surface of the inoculated agar. The plates were then incubated at 28 °C, and minimum inhibitory concentrations (MICs) were determined approximately 48 h post-incubation by identifying the lowest drug concentration.

### 2.3. Outcome Definition

The outcome at the last visit was considered for final analysis. The poor outcome was defined as visual acuity (VA) worse than counting fingers (CF), whereas the favorable outcome was defined as visual acuity (VA) of CF or better. In order to analyze conveniently, patients who underwent enucleation surgery until the end of the follow-up period were classified as having a poor outcome.

### 2.4. Statistical Analysis

The statistical analysis was conducted using SPSS (version 25.0; IBM, Armonk, NY, USA). Continuous variables are presented as medians along with their ranges, while categorical variables are presented as frequencies and percentages. Continuous variables were analyzed using Wilcoxon rank sum tests, while categorical variables were analyzed with Fisher’s exact tests. Cases with missing variables were excluded from the analysis of that variable. Differences were considered to be significant at *p* < 0.05.

## 3. Results

### 3.1. Clinical Features

In the current study, we reviewed the clinical features of 34 patients with culture-proven *P. lilacinum* from 1 January 2020, to 31 December 2024. A total of 17 men and 17 women were included; among them, the median age was 61.5 years (range from 4 to 76 years), with 17 right and 17 left eyes involved.

The most common risk factor was trauma. Thirteen (38.2%) patients had a history of ocular trauma, with nine cases involving vegetable matter or soil and one case involving insects. A history of prior ocular surgery was present in five (14.7%) patients. Namely, cataract surgery (n = 3), pterygium excision (n = 1), and eyelid squamous cell carcinoma surgery (n = 1). One (2.94%) patient had thyroid-associated ophthalmopathy and received systemic corticosteroid therapy. The remaining 15 (44.1%) patients had no history of ocular disease or surgery, and their risk factors remain unknown. Nine patients received topical or systemic corticosteroid treatment at the start of the symptoms, and the corticosteroid was stopped in all patients when fungal infectious disease was suspected. Among the patients who received corticosteroids, two had concurrent hypertension and one had concurrent diabetes. Additionally, there were six patients with hypertension only, one with diabetes only, and one with preexisting tuberculosis. Detailed information is shown in Table 1.

**Figure 1:**
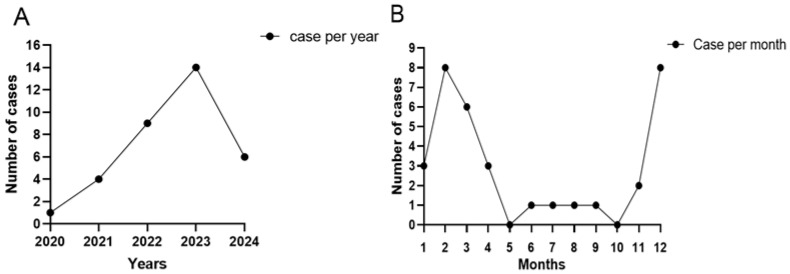
The distributions of years (**A**) and months (**B**) of recent 5 years for *P. lilacinum* oculomycosis.

The median duration between the onset of symptoms and microbiological diagnosis was 47.5 days (range from 13 to 210 days). The exact case numbers in the years 2020–2024 are shown in Figure 1A. The year with the largest number of cases in our center was 2023, with 14 cases, and the one with the least number of cases was 2020, with 1 case. A seasonal trend could be found in the cases of *P. lilacinum* oculomycosis in Figure 1B. The number of cases peaked in the winter of December to February with 19 cases (55.9%). In contrast, only four cases (11.8%) occurred in the hot months of May to October.

The visual acuity at presentation included seven patients with the best corrected visual acuity (BCVA) of 0.05 vision or better and one with 0.025 vision. The rest were counting fingers or worse. Most patients presented with symptoms of pain, redness, and visual loss. Corneal perforation was found in eight eyes.

The type of infection in our cases included 21 cases of fungal keratitis (61.8%), 7 cases of fungal endophthalmitis (20.6%), 4 cases of fungal keratitis with endophthalmitis (11.8%), and 2 cases of fungal keratoscleritis (5.9%).

### 3.2. Treatment and Outcome

Before a culture-positive *P. lilacinum* diagnosis, 24 (70.6%) patients were on empirical antifungal treatment, of which 20 patients were treated with voriconazole, three patients only with fluconazole, and one patient with natamycin. Voriconazole was started in 33 patients after *P. lilacinum* culture-positive results and antifungal susceptibility results were received. Of these, seven patients were treated only with topical voriconazole, while twenty-six patients received both topical and oral voriconazole, among whom eight also received intravenous treatment. Other antifungal drugs used in conjunction included fluconazole, amphotericin, natamycin, terbinafine, and itraconazole. Among the twenty-six patients who received systemic voriconazole treatment, three had their treatment of voriconazole discontinued or adjusted due to hepatic impairment.

Thirteen (38.2%) of the thirty-four patients underwent surgical interventions. Surgical procedures included pars plana vitrectomy (PPV) in five cases and penetrating keratoplasty in five cases, with one patient receiving penetrating keratoplasty combined with PPV. Two cases underwent enucleation.

Among the 31 cases with complete visual acuity records, the final BCVA included 16 cases with HM vision or worse, 3 with CF vision, 2 with 0.025 vision, and 10 with 0.05 vision or better (Table 2). The time to microbiological diagnosis in the favorable outcome group was 26 days, which is significantly shorter than that in the poor outcome group (*p* = 0.007). Moreover, the age and sex differences between the two groups did not reach statistical significance (Table 3).

### 3.3. Clinical Manifestations and Microbiological Identification

**Figure 2:**
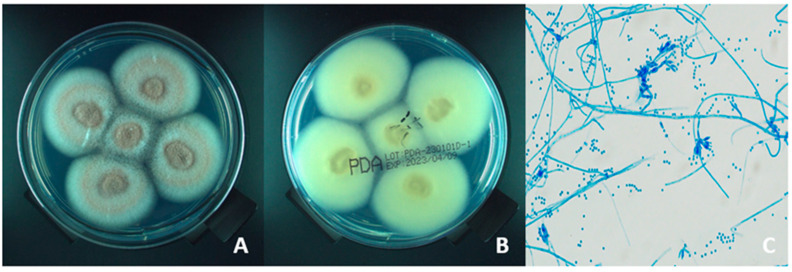
(**A**–**C**) Potato Dextrose Agar, incubated at 28 °C, displaying *P. lilacinum* colonies development after five days of culture. (**A**) Lilac, suede-like *P. lilacinum* colony with concentric ridges. (**B**) The back of the colony is yellowish-white. (**C**) Lactophenol cotton blue staining on a ×400 objective lens. Conidiophores are branched, featuring densely clustered whorls of phialides with swollen bases and a slender neck. Conidia are ellipsoidal to fusiform with a smooth wall.

Smear examination with fluorescence microscopy detected fungal elements in 76% (17/25) of corneal tissue samples, 60% (3/5) of aqueous fluid samples, and 50% (2/4) of vitreous body samples. Hypopyon was noted in 16 (47.1%) of the 34 eyes. Colonies on potato dextrose agar (PDA) exhibited rapid growth, initially appearing white before gradually turning lilac. The back of the colony is yellowish-white (Figure 2A,B). The lactophenol cotton blue wet mount was prepared and analyzed under optical microscope at 5 days of culture. The mount showed the conidiophore presents a broom branch and typical phialides with a distinct neck bearing conidia (Figure 2C). Altogether, the combination of macroscopic and microscopic features enabled the identification of the fungi as belonging to the genus *Purpureocillium*. At the same time, the isolates were performed the MALDI-TOF MS analysis and identified as *P. lilacinum*.

### 3.4. Antifungal Susceptivity

Table 4 presents the in vitro activities of voriconazole, caspofungin, and fluconazole against 34 *P. lilacinum* isolates. Fluconazole and caspofungin were inactive against *P. lilacinum*. The MIC of fluconazole was ≥256 mg/L for all tested isolates. The MIC of caspofungin was ≥32 mg/L. The median MIC for voriconazole was 0.25 mg/L (ranged from 0.064 to 24 mg/L). The voriconazole MIC for almost all isolates was ≤2 mg/L, except for one strain with MIC of 24 mg/L.

## 4. Discussion

A total of 34 patients (34 eyes) with culture-proven oculomycosis caused by *P. lilacinum* were reviewed. Our study showed that the number of cases peaked in the winter from December to February with 19 cases (55.9%). Trauma (38.2%) was the most common risk factor. Compared with the group of poor outcomes, the time to microbiological diagnosis in the group of favorable outcomes was significantly shorter (*p* < 0.05). Among the antifungal agents we tested, the isolates only demonstrated low MICs for voriconazole. Although most of our patients were treated with voriconazole, more than half of them still had a poor outcome.

In our study, a seasonal trend could be found in the cases of *P. lilacinum* oculomycosis. Nineteen cases (55.9%) occurred in the winter months from December to February, which aligns with previous reports that *P. lilacinum* infections clustered during autumn and winter [17]. Studies from northern India and southeastern China indicate that the incidence of fungal keratitis rises during the post-monsoon season and the harvest season [18,19]. Besides climatic factors and agricultural activities, the increased number of *P. lilacinum* oculomycosis cases in our series during cooler seasons may also be attributed to its relatively low optimal growth temperature [20,21]. Moreover, the number of *P. lilacinum* infections in our center increased from 1 case in 2020 to a peak of 14 cases in 2023, suggesting that there has been a growing incidence of *P. lilacinum* infections in recent years, highlighting the need for increased clinical awareness.

In our study, trauma (38.2%) was the most common risk factor, followed by prior ocular surgery (14.7%) and ocular diseases (2.94%), which is different from previous studies. For example, Pastor et al. reviewed the literature on *P. lilacinum* infection between 1964 and 2004, reporting that lens implantation (32.1%) was the most common risk factor [9]. Turner et al. reported that the primary risk factor is immunosuppression (81.25%) in Queensland, Australia [12]. In addition, Ali et al. reported that over 70% of the patients had a history of soft contact lens use in South Florida [11]. Many factors may have contributed to this discrepancy because the risk factors for oculomycosis vary depending on the region and environment. It is noteworthy that our study did not observe any instances of contact lens-associated infections. Chen et al. retrospectively reviewed 62 cases reported after 2009 and found that contact lenses (52%) were the most common risk factor in recent years [22]. The absence of contact lens-associated infections in our study, which contrasts with recent international reports, may reflect the specific demographics of our patient population and the continued prevalence of trauma as the primary route of inoculation in this region.

Early diagnosis and prevention strategies are crucial for effectively managing fungal keratitis [23]. Previous studies reported that patients with a shorter presentation had better visual outcomes [11]. Similarly, our study revealed that the favorable outcome group was more likely to have a shorter time to microbiological diagnosis (26 vs. 65 days, *p* < 0.05). This finding indicates that a delay in diagnosis is a poor visual prognostic factor. The clinical manifestations of *P. lilacinum* ocular infection are typically nonspecific and difficult to distinguish from other fungal infections.

The definitive diagnosis of *P. lilacinum* infections is dependent on the fungus identification using microbiological techniques. In recent years, MALDI-TOF mass spectrometry (MALDI-TOF MS) has been widely adopted by clinical laboratories worldwide as a powerful and efficient tool in the diagnosis of fungal keratitis, especially for cases involving rare or uncommon fungi [24]. Barker et al. found that compared with molecular diagnostic standards, there was 92.2% (71/77) agreement between the molecular and proteomic methods in identifying *Paecilomyces* spp. and *P. lilacinum* [25]. Moreover, in the study by Kuthan et al., the application of MALDI-TOF MS was able to accurately identify *P. lilacinum* 2 days earlier than conventional methods [26]. In our study, all 34 isolates were subjected to MALDI-TOF MS identification. It provides a rapid processing time and functions as a dependable diagnostic approach. We suggest that ocular specimens should be obtained and cultured for identification as early as possible. Early and accurate identification of the pathogen is important to enable early antifungal therapy.

Previous studies on *P. lilacinum* infections showed susceptibility to second-generation triazoles such as voriconazole and posaconazole. However, amphotericin B, natamycin, and first-generation triazoles like fluconazole or itraconazole showed poor in vitro activity against *P. lilacinum* [9,27,28]. In our study, all tested isolates demonstrated high MICs to fluconazole and caspofungin. Voriconazole showed good in vitro activity in most isolates with a median MIC of 0.25 mg/L, consistent with the previous studies. Voriconazole shows excellent penetration into the anterior chamber and vitreous, enabling therapeutic levels to be achieved via topical, oral, or intravenous without significant adverse effects on the retinal or corneal endothelium [29,30,31]. 97.1% of patients in our study received topical voriconazole or a combination of topical and systemic voriconazole therapies. However, several patients did not show a favorable clinical response with voriconazole treatment, suggesting the presence of refractory cases and need to consider alternative therapies. In a retrospective clinical study, posaconazole was evaluated as salvage therapy in patients with invasive aspergillosis who were proven to be refractory or intolerant to voriconazole. It indicated that the total success rate of posaconazole salvage therapy at day 60 was 72.2% [32]. Posaconazole showed the best in vitro activity against *P. lilacinum* [28,33]. Importantly, several case reports have documented refractory *P. lilacinum* keratitis patients resistant to voriconazole, whose infection was effectively controlled by the addition of posaconazole with no recurrence [34,35,36]. Notably, the isolate from Case 5 exhibited an unusually high voriconazole MIC of 24 mg/L. While rare, MIC values as high as 16 mg/L and 8 mg/L have been reported in previous studies [9,37], suggesting the potential emergence of *P. lilacinum* variants with reduced susceptibility to voriconazole. Clinically, this patient developed symptoms following ocular trauma caused by an insect. The patient received topical and oral voriconazole therapy for over five months, during which the infection was gradually controlled, achieving a final BCVA of 0.05. This case highlights the discrepancy often observed between in vitro susceptibility testing and in vivo therapeutic response. Previous studies have indicated that for certain fungal strains, no significant correlations were found between MIC values and clinical outcomes [38]. Therapeutic efficacy is likely influenced by multiple factors, such as the host immune status, the specific site of infection, and the timing of appropriate antifungal therapy [39]. Our study also demonstrated that a delay in diagnosis is a poor prognostic factor for visual outcome. Overall, voriconazole demonstrated good in vitro activity against *P. lilacinum*, which is currently the most commonly used antifungal agent for *P. lilacinum* oculomycosis. Posaconazole has shown potential in patients who respond poorly to voriconazole and warrants further evaluation of its efficacy.

Oculomycosis caused by *P. lilacinum* is a sight-threatening ocular infection and is always associated with poor visual outcomes. A previous review by Pastor showed that, from 1964 to 2004, enucleation occurred in 38% of the cases, and vision loss was noted in 25% [9]. However, Chen et al. found that the surgical rate in recent publications has been significantly lower than in previous ones (34% vs. 69%), probably because of the prevalent use of voriconazole [22]. Our study revealed that 38.2% of patients required surgical interventions, consistent with Chen’s study. However, 54.5% of patients still had a poor outcome; HM in 8 patients, LP in 2 patients, and NLP in 6 patients. Two patients underwent enucleation. These findings suggest that the visual outcomes of *P. lilacinum* oculomycosis are typically unfavorable, and enucleation is still required in severe, uncontrolled infections.

The current study also has a few inherent limitations. Due to the rarity of *P. lilacinum* oculomycosis, there is a relatively limited sample size. Furthermore, we only included cultured-positive cases, which could have underrepresented the overall etiological factors of *P. lilacinum* oculomycosis. Nevertheless, our study provides valuable data on the risk factors, clinical manifestations, and antifungal susceptibilities of *P. lilacinum* oculomycosis.

## 5. Conclusions

This study reviewed 34 cases of culture-proven *P. lilacinum* oculomycosis in southern China. Our findings highlight that the visual outcomes of this infection remain generally poor, with the major clinical challenge being the delay in diagnosis. Therefore, we strongly recommend prompt microbiological investigation for patients with suspected intraocular infection. In terms of treatment, in vitro susceptibility testing demonstrated that voriconazole exhibits potent activity against *P. lilacinum*. While voriconazole remains the first-line therapeutic choice, the therapeutic potential of newer triazoles warrants further investigation.

## Figures and Tables

**Table 1:** Clinical information of patients with *Purpureocillium lilacinum* oculomycosis.

Case No.	Age/Sex (Years)	Date (Year/Month)	Risk Factor	Systematic Disease and Immunosuppressed	Time to Diagnosis (Days)	Antifungal Drug	Initial BCVA	Final BCVA
1	51/M	2020/12	Trauma	Tuberculosis	50	VCZ, FCZ, NM, AMB	HM	0.05
2	75/M	2021/6	Cataract surgery	None	60	VCZ, ITZ, AMB	LP	HM
3	71/M	2021/12	Pterygium excision	HBP	45	VCZ, FCZ	LP	CF
4	76/F	2021/12	Trauma	HBP	30	VCZ, FCZ	HM	HM
5	59/F	2021/12	Trauma	HBP	43	VCZ, FCZ, NM	HM	0.05
6	67/F	2022/1	Trauma	None	20	VCZ, FCZ	CF	0.4
7	59/M	2022/1	Trauma	None	15	VCZ	CF	0.075
8	70/M	2022/2	Unknown	None	35	VCZ, FCZ	HM	HM
9	53/F	2022/2	Trauma	None	161	VCZ, FCZ, AMB	LP	NLP
10	65/M	2022/3	Unknown	None	57	VCZ, FCZ	NLP	NLP
11	50/F	2022/7	Unknown	Topical and systemic steroids	180	VCZ, FCZ, AMB	HM	0.16
12	64/F	2022/8	Trauma	None	23	VCZ, FCZ	HM	0.025
13	34/M	2022/11	Unknown	Diabetes, systemic steroids	31	VCZ, AMB	CF	HM
14	68/M	2022/12	Thyroid-associated ophthalmopathy	Systemic steroids	210	VCZ, AMB	0.05	NLP
15	4/M	2023/1	Unknown	Topical steroids	26	VCZ, AMB	LP	0.1
16	75/F	2023/2	Unknown	None	67	VCZ, FCZ, AMB	N/A	N/A
17	62/F	2023/2	Trauma	None	67	VCZ, FCZ	CF	0.06
18	75/M	2023/2	Unknown	HBP	21	VCZ	0.075	0.025
19	57/M	2023/2	Unknown	HBP, Topical and systemic steroids	150	VCZ, AMB	0.025	LP
20	51/F	2023/2	Unknown	None	38	VCZ, FCZ, AMB	CF	CF
21	46/F	2023/2	Unknown	None	16	VCZ, FCZ, AMB	LP	HM
22	69/M	2023/3	Eyelid squamous cell carcinoma surgery	HBP	66	N/A	HM	EN
23	43/M	2023/3	Trauma	None	132	VCZ, FCZ, AMB	HM	NLP
24	75/F	2023/3	Unknown	HBP	97	VCZ, FCZ	LP	EN
25	59/F	2023/4	Unknown	Topical and systemic steroids	210	VCZ, AMB	0.05	LP
26	75/F	2023/4	Unknown	None	186	VCZ, AMB	NLP	NLP
27	72/F	2023/11	Trauma	Diabetes	64	VCZ	LP	HM
28	49/M	2023/12	Unknown	None	24	VCZ	0.16	0.075
29	62/M	2024/3	Trauma	None	30	VCZ	HM	CF
30	63/F	2024/4	Trauma	None	21	VCZ, FCZ, AMB	0.075	0.12
31	57/F	2024/3	Cataract surgery	HBP, Topical and systemic steroids	13	VCZ, TRB	CF	NLP
32	58/F	2024/9	Unknown	None	13	VCZ, NM, AMB	0.075	0.4
33	61/M	2024/12	Cataract surgery	Topical and systemic steroids	54	VCZ, FCZ	HM	HM
34	53/M	2024/12	Trauma	Topical steroids	77	VCZ, FCZ	0.05	HM

F, female; M, male; HBP, high blood pressure; AMB, amphotericin; FCZ, fluconazole; NM, natamycin; VCZ, voriconazole; TRB, terbinafine; BCVA, best-corrected visual acuity; CF, counting fingers; HM, hand motion; LP, light perception; NLP, no light perception. N/A, not available; EN, enucleation.

**Table 2:** Comparison of the final BCVA with the initial BCVA.

	Initial BCVA (n, %)	Final BCVA (n, %)
≧0.05	7 (22.6)	10 (32.3)
>CF − 0.05	1 (3.2)	2 (6.5)
CF	6 (19.4)	3 (9.7)
HM	9 (29.0)	8 (25.8)
LP	6 (19.3)	2 (6.5)
NLP	2 (6.4)	6 (19.4)

n = 31, as two patients underwent enucleation, and one patient’s BCVA data were not available. CF, counting fingers; HM, hand motion; LP, light perception; NLP, no light perception.

**Table 3:** Visual outcome and clinical background of 33 eyes.

Characteristic	Favorable Outcome (n = 15)	Poor Outcome (n = 18)	*p* Value
Age, median year (range)	59 (4–75)	63 (34–76)	0.337
Gender,			0.732
male (%)	7 (46.7%)	10 (55.6%)	
female (%)	8 (53.3%)	8 (44.4%)	
Risk factor, no. (%)			0.339
Trauma	8 (53.3%)	5 (27.8%)	
prior ocular surgery	1 (6.7%)	4 (22.2%)	
Ocular disease	0 (0%)	1 (5.6%)	
Unknown	6 (40.0%)	8 (44.4%)	
Immunosuppressed, no. (%)	2 (13.3%)	7 (38.9%)	0.134
Time to diagnosis, median day (range)	26 (13–180)	65 (13–210)	0.007

n = 33, as one patient’s BCVA data were not available.

**Table 4:** In vitro antifungal susceptibilities of *Purpureocillium lilacinum*.

	Antifungal Drugs (MICs mg/L)		
Case No.	Voriconazole	Fluconazole	Caspofungin
1	0.25	≥256	≥32
2	0.5	≥256	≥32
3	0.094	≥256	≥32
4	0.25	≥256	≥32
5	24	≥256	≥32
6	0.25	≥256	≥32
7	0.38	≥256	≥32
8	0.19	≥256	≥32
9	0.19	≥256	≥32
10	0.094	≥256	≥32
11	0.25	≥256	≥32
12	0.5	≥256	≥32
13	0.064	≥256	≥32
14	0.25	≥256	≥32
15	0.094	≥256	≥32
16	0.19	≥256	≥32
17	0.25	≥256	≥32
18	0.5	≥256	≥32
19	0.25	≥256	≥32
20	1	≥256	≥32
21	0.125	≥256	≥32
22	0.5	≥256	≥32
23	0.125	≥256	≥32
24	0.094	≥256	≥32
25	0.25	≥256	≥32
26	0.19	≥256	≥32
27	0.25	≥256	≥32
28	1	≥256	≥32
29	0.5	≥256	≥32
30	0.25	≥256	≥32
31	0.5	≥256	≥32
32	2	≥256	≥32
33	2	≥256	≥32
34	0.5	≥256	≥32

## Data Availability

The original contributions presented in this study are included in the article. Further inquiries can be directed to the corresponding authors.
